# VIOLENCE AGAINST CHILDREN AND ADOLESCENTS: NOTIFICATION AND ALERT IN TIMES OF PANDEMIC

**DOI:** 10.1590/1984-0462/2021/39/2020267

**Published:** 2020-10-28

**Authors:** Vanessa Borges Platt, Jucélia Maria Guedert, Elza Berger Salema Coelho

**Affiliations:** a Universidade Federal de Santa Catarina, Florianópolis, SC, Brazil.

**Keywords:** Domestic violence, Pandemics, Coronavirus infections, Child, Adolescent, Violência doméstica, Pandemias, Infecções por coronavírus, Criança, Adolescente

## Abstract

**Objective:**

Social isolation is currently identified as the best way to prevent the infection by the new coronavirus. However, for some social groups, such as children and adolescents, this measure carries a contradiction: the home, which should be the safest place for them, is also a frequent environment of a sad aggravation: domestic violence. This study aims to evaluate the notifications of interpersonal/self-inflicted violence available in the Information System for Notifiable Diseases in the State of Santa Catarina (southern Brazil), for the juvenile age group, before and during the new coronavirus pandemics.

**Methods:**

Cross-sectional, descriptive study of violence against children and adolescents (from 0 to 19 years) notified by health professionals by completing and entering the occurrence in the Information System for Notifiable Diseases of the State of Santa Catarina in 11 weeks in which the social isolation measure was instituted as mandatory, comparing with the same period before this measure.

**Results:**

During the study period, 136 municipalities in Santa Catarina made 1,851 notifications. There was a decrease of 55.3% of them in the isolation period, and the difficulties encountered in seeking protection and assistance institutions were listed.

**Conclusions:**

The society needs to be aware of possible cases of violence in the children and adolescent population. It is important to provide accessible, effective, and safe ways for complaints and notifications, as well as a quick response to the cases, aiming at protecting victims and minimizing damages to prevent the perpetuation of the violence.

## RESUMO

**Objetivo:**

O isolamento social é identificado, no momento, como a melhor forma para evitar o contágio pelo novo coronavírus. Porém, para alguns grupos sociais, como crianças e adolescentes, essa medida carrega uma contradição: o lar, que deveria ser o local mais seguro para eles, é também um ambiente frequente de um triste agravo, a violência doméstica. Este estudo visou avaliar e comparar as notificações compulsórias de violências interpessoais/autoprovocadas disponíveis no Sistema de Informação de Agravos de Notificação do Estado de Santa Catarina, pré e pós-pandemia do novo coronavírus.

**Métodos:**

Estudo transversal, descritivo e analítico das violências contra crianças e adolescentes (de 0 a 19 anos de idade completos) notificadas pelos profissionais de saúde mediante o preenchimento e a inserção das ocorrências no Sistema de Informação de Agravos de Notificação do Estado de Santa Catarina, no período de 11 semanas em que foi instituída como obrigatória a medida de isolamento social, comparando tais eventos com os de igual período anterior a essa medida.

**Resultados:**

No período estudado, 136 municípios catarinenses realizaram 1.851 notificações. Houve diminuição de 55,3% destas no período de isolamento, listando-se possíveis dificuldades encontradas para a procura de instituições de proteção e assistência.

**Conclusões:**

Alerta-se para a necessidade de a sociedade estar atenta para a suspeita e evidência dos casos de violência na população infantojuvenil, e ressalta-se a importância de que sejam propiciadas formas acessíveis, eficazes e seguras, como incentivo para as denúncias, a notificação e o rápido atendimento dos casos, visando à proteção das vítimas, à minimização dos danos e, assim, ao impedimento da perpetuação da violência.

## INTRODUCTION

On March 11, 2020, the World Health Organization (WHO) recognized that COVID-19 disease, caused by the new coronavirus (SARS-CoV-2), has taken pandemic proportions.^1^ This unknown disease, with no possibility, until now, of pharmacological treatment or vaccine control, imposed the guidance, by the entity, of social isolation as the only way to contain its spread.

Since the first cases identified in Brazil, the population has turned its attention to the devastating numbers of contaminated people and deaths, the adequacy with the ways to avoid contagion and the sanitary obligation of social isolation. As a result, daily activities of children and adolescents outside their home were prohibited, such as attending classes, circulating in public environments, and even hanging out with friends, restricting the social space of individuals to the home environment.

Despite laws and advances in the development of assistance and care strategies, the numbers on violence in pre-pandemic Brazil were already worrying.

Data from the Informatics Department of the Unified Health System (DATASUS) reveal that in 2017 there were 126,230 cases of violence against children and adolescents up to the age of 19 (corresponding to 42% of the total cases reported that year). The record of 21,559 deaths from external causes, accidents, and violence up to the age of 19 shows that many did not resist the abuse. Of these cases, a quarter died before the age of 10, and more than 10% (2,309 children) were up to 4 years old.^2^

In 2019, the Dial Human Rights (Dial 100), a telephone tool provided by the Brazilian government to receive, analyze, and forward complaints of human rights violations, including cases of violence, revealed 159,063 complaints of maltreatment - an increase of 15% compared to 2018. Of these complaints, 86,837 were violence against children and adolescents (55%), distributed as follows: 38% linked to negligence, 23% to psychological violence, 21% to physical violence, 11% to sexual violence, 3% to exploitation/child labor, and 3% associated with other violent injuries. The most frequent place of occurrences was the victim's home.^3^

In the state of Santa Catarina, from 2015 to 2019, 65,672 notifications were registered in the Information System for Notifiable Diseases (SINAN). Of these, 38.4% occurred in the age group of children and adolescents.^4^

Intrafamily violence is difficult to unravel, as it occurs in the private sphere, in the domestic environment, within homes and is protected by the law of silence, by the fear, and impunity of its agents - people who should support and protect children and adolescents. This violence covers five types: physical, sexual, psychological, neglect, and specific forms, which are expressed in the forms of Münchhausen syndrome, chemical violence, and filicide.^5^ Paradoxically, the home, the safest environment for people to be protected from contagion by the new coronavirus, while there is no vaccine available, can be the most unsafe place for many children and adolescents.^6^ The concern about a regrettable and well-known wound in our society emerges from this situation: domestic violence against children and adolescents - often at home and perpetrated by family members.

This study aimed to present data on compulsory notifications on cases of violence against children and adolescents in the state of Santa Catarina, in the months after the emergence of the new coronavirus, and how the establishment of sanitary measures of social isolation influenced the increase in domestic violence against children and adolescents when comparing this information to that of the pre-pandemic, to alert health professionals, public institutions, and society to the need to reinforce actions to prevent injuries, protection, and adequate care for victims.

## METHOD

This is a cross-sectional, descriptive, and analytical study of violence against children and adolescents (aged 0 to 19 years old) notified by health professionals, by filling out and inserting into the SINAN the Notification Forms for Individual Investigation of Interpersonal/Self-inflicted Violence,^7^ between January and May 2020, in the state of Santa Catarina. The age limit is because the WHO defines this age range for children and adolescents.^8^

Santa Catarina has 295 municipalities, distributed over 95,730,921km^2^. According to the Brazilian Institute of Geography and Statistics (IBGE), in 2012, the Brazilian state was the 11th in size of the population, with 6,248,436 inhabitants. The estimated population in 2019 was 7,164,788 inhabitants, with 971,034 being the population of children and adolescents. ^9^

The study analyzed the data using descriptive statistics.

## RESULTS

From January 1 to May 31, 2020, 136 of the 295 municipalities in Santa Catarina made 1,851 notifications in SINAN of suspected or confirmed cases of interpersonal or self-inflicted violence in the 0 to 19 age group. These events were characterized as: neglect or abandonment, physical, psychological, sexual, and other violence, and child labor (Table 1).^10^

**Table 1:** Typology of notifications of child and adolescent violence in the COVID-19 pandemic, Information System for Notifiable Diseases, Santa Catarina*, between January and May 2020.

Age group (years)	Neglect/abandonment		Physical violence		Sexual violence		Psychological violence	
	n	%	n	%	n	%	n	%
<1	184	32.1	33	6.0	11	2.7	6	3.0
1 to 4	241	42.0	33	6.0	70	17.2	18	9.1
5 to 9	70	12.2	43	7.8	112	27.4	26	13.1
10 to 14	68	11.8	122	22.3	152	37.3	54	27.3
15 to 19	11	1.9	318	57.9	63	15.4	94	47.5
Total*	574	100	549	100	408	100	198	100

*120 notifying municipalities.

Source: Directorate of Epidemiological Surveillance of Santa Catarina.^10^

At the time of data collection for this study, only 46% of the municipalities had included cases of violence in SINAN. The data request was sent to all municipal health departments in the state of Santa Catarina. The response of less than half of them may be due to the need for restructuring health facilities and adapting their facilities to the service demands of the pandemic.

Table 2 shows the percentage differences in the number of notifications of child and adolescent violence accumulated in the weeks before and after the decree that established the measures of social isolation in Santa Catarina.

**Table 2:** Child and adolescent violence notifications accumulated for weeks before and after adopting social isolation measures in Santa Catarina*.

Age group (years)	Period				Total
	January 1^st^ to March 15^th†^		March 16^th^ to May 31^st‡^		
	n	%	n	%	
<1	137	7.4	87	4.7	224
1 to 4	211	11.4	125	6.8	336
5 to 9	135	7.3	81	4.4	216
10 to 14	266	14.4	147	7.9	413
15 to 19	443	23.9	219	11.8	662
Total	1,192	64.4	659	35.6	1,851

*136 notifying municipalities; ^†^period of 11 weeks before the guidance on social isolation; ^‡^period of 11 weeks after the guidance of social isolation.

Source: Directorate of Epidemiological Surveillance of Santa Catarina.^10^

## DISCUSSION

For 30 years, the Child and Adolescent Statute (ECA), under Law No. 8,069/90, made children and adolescents “subjects of law” in Brazil. It delegates to society the duties to protect and care for these developing Brazilian citizens and emphasizes the obligation to ensure full compliance with the rights necessary to promote their full potential, removing any form of oppression or discrimination. In its Article 13, ECA determines the obligation to report all cases of suspected or confirmed physical punishment, cruel or degrading treatment, and maltreatment of children to the local child protection councils, without damage to other legal measures.^11^

In 2001, the notification of suspected or confirmed cases of abuse against children became mandatory and directed to municipal epidemiological surveillance and the child protection council.^12^ In 2011, this notification became computerized, through compulsory filling for all health services, both public and private, of the SINAN Notification Form/Individual Investigation of Interpersonal/Self-inflicted Violence.^13^

Data from social organizations and non-governmental institutions published in the media report an increase in violence against children and adolescents in the pandemic, such as the 7.4% increase in the Federal District,^14^ 8.5% in Paraná,^15^ 73% in Rio Grande do Sul^16^, and 32% in Pernambuco.^17^ SaferNet, a civil association governed by private law, with national operations, focused on the promotion and defense of human rights on the internet in Brazil, in partnership with the National Committee to Combat Sexual Violence against Children and Adolescents, registered a 108% increase in complaints on child pornography during the country's pandemic; in April 2020 alone, there were 9,995 complaints.^18^

The state of Santa Catarina has provided data on the number of cases of domestic violence against children and adolescents since the emergence of the COVID-19 pandemic.

Based on SINAN notifications from 136 municipalities in Santa Catarina that released data (out of a total of 295) for 2020, there was a progressive decrease in the total number of notifications since the beginning of social isolation, the absolute numbers in January, February, March, April, and May are, respectively, 469, 506, 434, 273, and 169 (Table 2).^10^ When comparing January with April, for example, we found a 42% decrease in the number of notifications of child and adolescent violence, the percentage of which is higher (64%), if we compare January to that of May.

Does this data mean that violence has decreased? We believe it has not. Several factors can justify the significant reduction in notifications. The need for restructuring and adapting health services to the pandemic reality, directing employees and health units for the exclusive care of cases of acute respiratory syndromes, and overload of the teams of health workers due to the increased demand for care may have made it difficult for users to access services usually available to the population. Added to this, we have the interruption of public transport services, which imposed difficulties in traveling and accessing health services. The fear of contamination, the social isolation imposed, the limitations for leaving home, added to the financial difficulties resulting from the pandemic, may have constituted barriers to reporting violence and seeking assistance and the consequent decrease in notifications.^14^

For many women, the health measures adopted to cope with the new coronavirus demanded more domestic work, greater care for children, away from daycare centers and schools, besides greater assistance to the elderly and sick family members. It is inferred that movement restrictions, financial limitations, and generalized insecurity encourage abusers, giving them a greater sense of power and control.^19^

Confinement also brings different changes in the routine of family members, leading to stress, which, if not well conducted, can lead to consequences for the entire family dynamics. These consequences can affect the physical and mental health of children and adolescents, especially young children, who do not have the necessary tools to adjust to stress or to overcome it, which can make it especially harmful.

Family stress is closely associated with uncertainty/insecurity in the near future, the possibility of falling ill and experiencing the illness of a family member/loved one, fear of not getting adequate access to health, not promising news in the media, economic problems related to job loss and/or reduction of monthly family income. Added to these facts is the increased time spent with children and adolescents, now almost 24 hours, both due to the need for social isolation and the closing of schools, parks, and leisure areas in the condominiums.

Stress is also associated with the absence of other components of the family support network, such as the interaction with grandparents, uncles, neighbors, domestic workers, and even institutions such as churches and social projects. This tension experienced and expressed by parents is reflected in children and adolescents, who start to adopt the same behavior: tension, demotivation, and aggression, which can be intensified by the excessive time on screen.

This whole range of factors favors a violent domestic environment, which, associated with the distance from the protection agencies, the fear of losing the only provider of the family, of not being able to leave the house, for example, to stay at their parents' house (grandparents of the children), even because of the fear of rupturing the relationship, thus enabling the maintenance of the pact of silence inside the house, making everyone vulnerable to suffering violence.^20^

Family violence has a strong historical-cultural connotation: parents who were educated in a violent manner reproduce this form of education, resorting to physical and psychological violence, manifested by beatings, hair pulling, spanking, shouting, and the most diverse threats as a way to impose discipline to their children.

The protective role of the school is recognized as paramount, identifying in the teacher often the confidant, the professional with a watchful eye on the students under their care, the one who can be the trigger for the protection and care network and guarantee the rights of children and adolescents. However, according to the United Nations Educational, Scientific and Cultural Organization (Unesco), about 1.5 billion children and adolescents worldwide are out of school because of the closure of educational institutions as an initiative to contain cases of COVID-19.^21^ Therefore, children no longer have an important space to manifest and expose the violence suffered.

Still, much of the state's child protection structure, such as child protection councils and police stations, is providing virtual assistance only. Additionally, public transport is absent in many regions and greater consumption of alcohol and other drugs, as well as psychoactive medications.^19^

A recent publication was assertive when it was titled “The pandemic paradox”, as this pandemic creates a paradox concerning security at home: a place where we should be protected and safe is where violence occurs for the most vulnerable groups. The authors emphasize that we must all pay attention to this issue: “Governments around the world have asked all of us to participate in the fight against COVID-19 by staying at home, but it is also important to pay critical attention to what this means for many women and children”.^6^

Given this, specific and regional regulations were developed, initiatives to be thought and reproduced by states and municipalities, such as the National Council for the Rights of Children and Adolescents, dictating recommendations for the full protection of children and adolescents during the COVID-19 pandemic,^22^ and those of the state of Minas Gerais, through Law No. 23,643, of 2020,^23^ which requires residential condominiums to inform security agencies about episodes or signs of domestic violence in their common and private facilities, and the Law No. 23,644, also from 2020,^24^ which deals with the registration of this type of occurrence through the virtual police station system, during the pandemic.

On June 10, the National Council of Justice and the Association of Brazilian Magistrates launched the Red Flag Against Domestic Violence campaign. The initiative focuses on helping women in situations of violence to ask for help in pharmacies in the country, offering a silent channel of communication and assistance: with a red X in the palm of the hand, which can be done with a pen or even lipstick, the victim indicates she is in a situation of violence. With the woman's name and address in hand, pharmacies and drugstore attendants who join the campaign should immediately call 190 and report the situation. At the time of its launch, the project had a partnership with 10,000 pharmacies and drugstores across the country.^25^

In the same direction, the Military Police of Santa Catarina and the National Council of General Commanders of the Military Police and the Military Fire Brigade joined forces, dispatched and disseminated, through the internal network, normative instruction with the addition of standard operating procedures for the specific handling of incidents involving domestic violence against women.^26^

In the same state, Technical Note 012/2020, from the State Department of Health, dated May 19, 2020, deals with measures to combat domestic violence in the context of the COVID-19 pandemic. It warns of the importance of notifying suspected or confirmed cases and referring them as soon as possible to protection agencies and multiprofessional care as recommended in the care protocols. The note also lists suggestions for protective actions for victims and informs contact phones of entities in the protection network.^27^

Law no. 14.022 was recently published in the Federal Official Gazette of Brazil (Section 1, p. 3, of July 8, 2020), which ensures the full functioning, during the COVID-19 pandemic, of entities assisting women, children, adolescents, the elderly, and citizens with disabilities who are victims of domestic or family violence. According to the law, assistance to victims is considered an essential service and cannot be interrupted while the state of public calamity caused by the new coronavirus lasts.^28^

We chose to study data on domestic violence in children and adolescents under COVID-19, considering the current concern of society for the risk of its intensification during home confinement imposed by the pandemic. The small number of municipalities that provided the notification data and the short period analyzed can be mentioned as limitations of this study. Nonetheless, we understand that these limitations are minimized by the relevance and urgency of the topic.

It is possible to conclude that the reduction in the number of complaints of violence against children and adolescents does not encourage, nor does it seem to reflect a decrease in the incidence of this condition. On the contrary, it can demonstrate difficulties that people may be facing in making the complaints and using existing social resources to care for the victims. Initiatives, although specific, are understood as beneficial, which warn of the need for attention to the problem. In this pandemic moment, with the confinement of children in potentially violent homes and spaces, it is essential that their surroundings and the whole society be alert to the suspicion and evidence of cases of violence and that accessible, effective, and safe ways are provided so that complaints, notification, and prompt response to cases occur, aiming at protecting victims, minimizing damage and, thus, preventing the perpetuation of violence.
